# Accelerated calibrationless parallel transmit mapping using joint transmit and receive low-rank tensor completion

**DOI:** 10.1002/mrm.28880

**Published:** 2021-07-01

**Authors:** Aaron T. Hess, Iulius Dragonu, Mark Chiew

**Affiliations:** 1 Oxford Centre for Clinical Magnetic Resonance Research (OCMR), University of Oxford, Oxford, United Kingdom; 2 Siemens Healthcare Limited, Frimley, United Kingdom; 3 Wellcome Centre for Integrative Neuroimaging, FMRIB, Nuffield Department of Clinical Neurosciences, University of Oxford, Oxford, United Kingdom

**Keywords:** autocalibration, calibration-free, calibrationless, cardiac MRI, low rank, parallel transmit, self-calibration, tensor completion, transmit mapping, ultrahigh field

## Abstract

**Purpose:**

To evaluate an algorithm for calibrationless parallel imaging to reconstruct undersampled parallel transmit field maps for the body and brain.

**Methods:**

Using a combination of synthetic data and in vivo measurements from brain and body, 3 different approaches to a joint transmit and receive low-rank tensor completion algorithm are evaluated. These methods included: 1) virtual coils using the product of receive and transmit sensitivities, 2) joint-receiver coils that enforces a low rank structure across receive coils of all transmit modes, and 3) transmit low rank that uses a low rank structure for both receive and transmit modes simultaneously. The performance of each is investigated for different noise levels and different acceleration rates on an 8-channel parallel transmit 7 Tesla system.

**Results:**

The virtual coils method broke down with increasing noise levels or acceleration rates greater than 2, producing normalized RMS error greater than 0.1. The joint receiver coils method worked well up to acceleration factors of 4, beyond which the normalized RMS error exceeded 0.1. Transmit low rank enabled an eightfold acceleration, with most normalized RMS errors remaining below 0.1.

**Conclusion:**

This work demonstrates that undersampling factors of up to eightfold are feasible for transmit array mapping and can be reconstructed using calibrationless parallel imaging methods.

## 1 Introduction

Parallel transmit (pTx) technology mitigates transmit field heterogeneity^1,2^; accelerates spatial RF pulses^3,4^; and lowers specific absorption rate deposition,^5–7^ which is achieved by driving multiple (parallel) transmit coils with subject, target, and pulse-specific amplitudes and phases. To generate subject-specific pulse designs, the transmit fields are measured for each subject and transmit channel, a time-consuming process in cardiac imaging, for example.^8^ This work investigates the acceleration of pTx field mapping for streamlining the use of pTx in MR imaging.

The minimum requirement for field mapping is to characterize the relative magnitudes and phases of the transmit channels (relative maps). Relative maps are analogue to coil sensitivities in parallel imaging and are measured by transmitting in different transmit configurations, usually 1 channel at a time, while acquiring data on all receive channels simultaneously.^9–11^ Relative mapping is only valid when the excitation flip angle is both large enough to generate sufficient signal (greater than the noise) and small enough that the signal is proportional to B1+.^12^ Whereas this requires fewer images than absolute B1+ mapping,^10^ it still typically results in a scan time cost that scales with the number of transmit channels.

As demonstrated by Padormo et al., the same k-space calibration data can be used to estimate both receive sensitivities and transmit sensitivities (precise RF inference from multiple observations [PRIMO]^13^). In the heart, gating to cardiorespiratory motion increases the measurement time by at least fivefold for mapping transmit fields (assuming a respiratory efficiency of 50% and cardiac acquisition window of 400 ms). Either multiple breathholds or freebreathing using self-gating^14^ can be used to cover the whole heart, which takes about 3 and 6 min, respectively.

To reduce measurement times, accelerated imaging techniques can be used to recover pTx field maps from under-sampled (k-space) measurements. Accelerating the pTx field calibration will both increase the scan time available for diagnostic imaging and streamline the pTx adjustments. In order to recover missing k-space data, a calibrationless parallel imaging approach can be used, for example, with prior knowledge about the low-rank Hankel (or block-Hankel) structure of matrices formed from local k-space neighborhoods.^15–18^ The simultaneous autocalibrating and k-space estimation (SAKE)^17^ method uses a projection-onto-sets algorithm with singular value thresholding to recover missing k-space data by alternating between enforcing data consistency and low-rank Hankel matrix structure without the need of any fully sampled calibration region. Simultaneous autocalibrating and k-space estimation (SAKE), and related methods such as Low-rank modeling of local k-space neighborhoods with parallel imaging (P-LORAKS)^15,16^ or annihilating filter based low-rank Hankel matrix approach (ALOHA),^19^ exploit redundancies that are present when Hankel matrices are combined across multiple coils. This enables a low-rank representation of the data to effectively constrain undersampled data recovery. In fact, these approaches are also similar to methods such as parallel imaging using eigenvector maps (ESPIRiT)^20^ and PRIMO,^13^ which form very similar low-rank Hankel-structured matrices, except that these latter methods work on fully sampled data. A comprehensive review of low-rank Hankel methods in MR image reconstruction can be found in Ref. 21.

In this work, we investigate the extension of these calibrationless parallel imaging approaches to recover undersampled relative transmit field maps using structured low rank tensor completion. We build on previous work that leverages image redundancy across multiple dimensions or encodings, for example.^22–24^ In this work a multi-dimensional tensor representation of both transmit and receive field dimensions is evaluated. This approach exploits varying undersampling patterns across different transmit encodings and enables information sharing across these encodings. We investigate, in simulations and experimental data in the body and the brain, the acceleration factors achievable with this approach and demonstrate that undersampling factors of up to 8× are feasible with our proposed method.

## 2 Theory

### 2.1 Matrix structure

Simultaneous autocalibrating and k-space estimation and the subspace identification part of ESPIRiT^20^ take k-space data and through the application of a kernel transform form a block-Hankel matrix representation. Subspace identification is performed through low-rank reconstruction of missing samples (simultaneous autocalibrating and k-space estimation) or SVD-based singular value truncation (ESPIRiT). PRIMO^13^ extends ESPIRiT to include transmit (Tx) sensitivities, which is achieved by concatenating the transmit and receive sensitives along different axes of the block-Hankel matrix. The time interleaved acquisition of modes (TIAMO)^25^ method harnesses transmit sensitives in the image reconstruction using different transmit modes (sensitives) as virtual receive channels, which results in a number of virtual receiver channels that are the product of receive channels and transmit modes. We refer to these methods below as virtual coil (VC) for the latter and PRIMO for the former.

Here we consider the VC and PRIMO representations to be special cases of a block-Hankel tensor formed from the multi-dimensional data *𝒟* ∈ ℂ^*N_kx_* ×*N_ky_* ×*N_Rx_* ×*N_Tx_*^, where the fourth-order tensor (or multi-dimensional array) *𝒟* is transformed to another fourth-order tensor *ℋ* ∈ ℂ^*N*_1_ ×*N*_2_ ×*N_Rx_* ×*N_Tx_*^ with block-Hankel frontal slices. That is, for each transmit and receive pair, the 2D k-space is transformed to a 2D block-Hankel matrix for which the “frontal slices” refers to the fact that the block-Hankel matrix is defined by the first 2 indices of the tensor.

**Figure 1:**
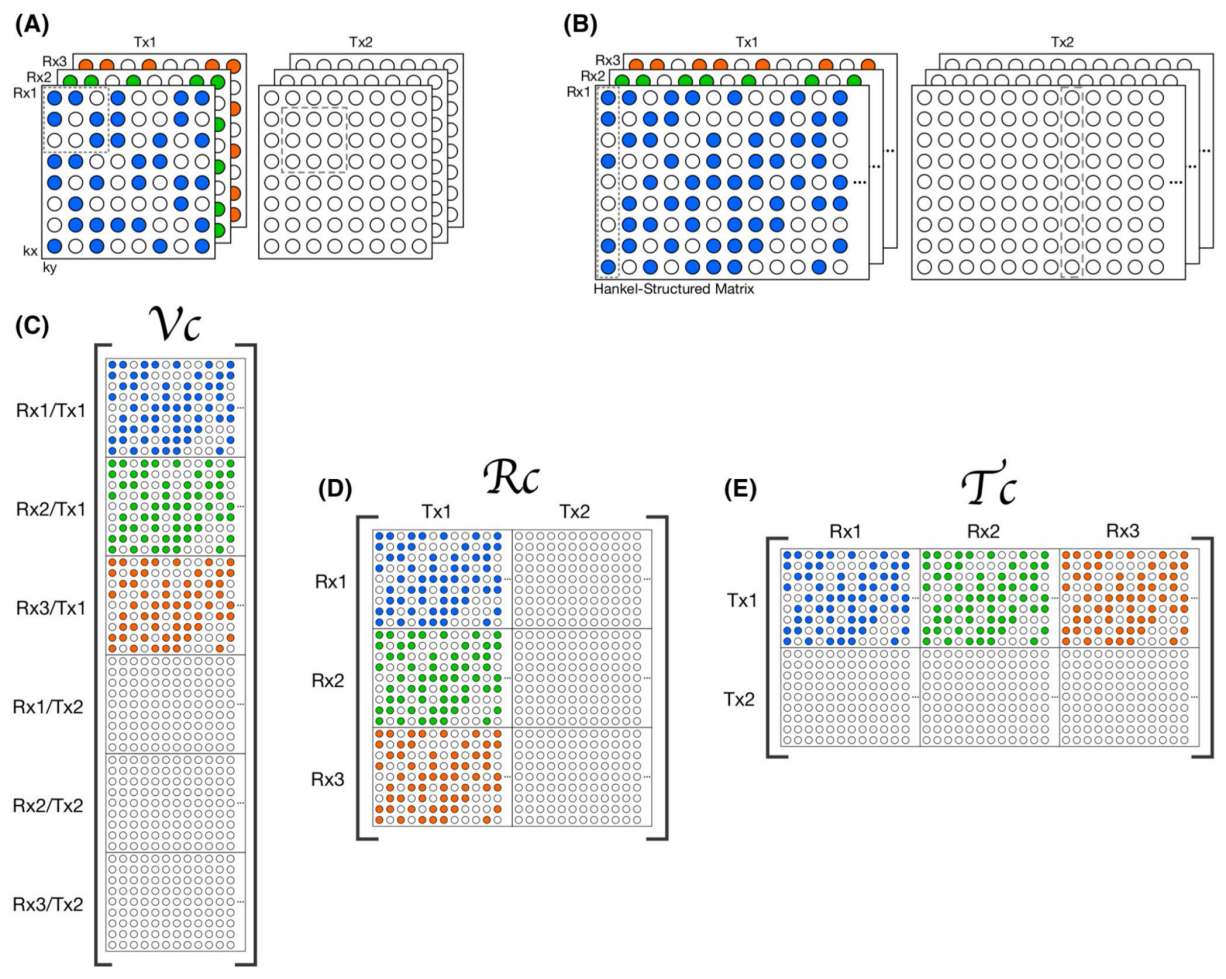
Diagram showing how (A) a multi-dimensional k-space dataset with dimensions kx, ky, Rx, and Tx can be transformed to (B), a fourth-order tensor with block-Hankel frontal slices. Colored circles provide a schematic representation of undersampling across k-space and Tx channels and illustrate how 2D k-space kernels in (A) are mapped to column vectors in (B). (C-E) show different matricizations or unfoldings of the tensor, corresponding to: (C) the *𝒱_𝒞_*, (D) the PRIMO-style *ℛ_𝒞_*, and (E) the *𝒯_𝒞_*. The proposed TxLR method uses low-rank constraints on both *ℛ_𝒞_* and *𝒯_𝒞_* unfoldings of the tensor. PRIMO; precise RF inference from multiple observations; R_C_, receive vertical concatenation matrix; T_C_, transmit vertical concatenation matrix; Tx, transmit; TxLR, transmit low rank; Rx, receive; V_C_, virtual coil matrix

The linear operator:

(1) $$T:{\mathbb{C}}^{N_{kx}\;\times N_{ky}\times N_{Rx}\times N_{Tx}}\to {\mathbb{C}}^{N_{1}\times N_{2}\times N_{Rx}\times N_{Tx}},$$

is what maps the k-space data corresponding to any given transmit and receive k-space into the block-Hankel *N*_1_ × *N*_2_ matrix by rastering over k-space with a kernel of dimension *N*_1_ = *m* × *n* (eg, *N*_1_ = 9 for a 3 × 3 kernel), resulting in *N*_2_ = (*N_kx_* − *m* + 1) · (*N_ky_* − *n* + 1) distinct kernel vectors. This is done for every transmit and receive pair, which results in the tensor *ℋ* =*T* (*𝒟*), as demonstrated in Figure 1.

We considered 3 different unfoldings of this tensor:

Virtual coils (VC): From the tensor *ℋ*, we can construct the matrix corresponding to the interpretation of every transmit and receive pair as a unique receive map by applying a particular unfolding of the tensor

(2) $$U_{0}:{\mathbb{C}}^{N_{1}\times N_{2}\times N_{Rx}\times N_{Tx}}\to \mathbb{C}\left(N_{1}\cdot N_{Rx}\cdot N_{Tx}\right)\times \left(N_{2}\right),$$

such that *𝒱_𝒞_* = *U*_0_ *ℋ* (Figure 1C).

Receive concatenation: Alternatively, we can construct a matrix corresponding to the *calibration matrix* in the PRIMO relative transmit mapping approach by applying the following unfolding of the tensor

(3) $$U_{2}:{\mathbb{C}}^{N_{1}\times N_{2}\times N_{Rx}\times N_{Tx}}\to {\mathbb{C}}^{\left(N_{1}\cdot N_{Rx}\right)\times \left(N_{2}\cdot N_{Tx}\right)},$$

such that *ℛ_𝒞_* = *U*_2_ *ℋ* (Figure 1D).

**Figure 2:**
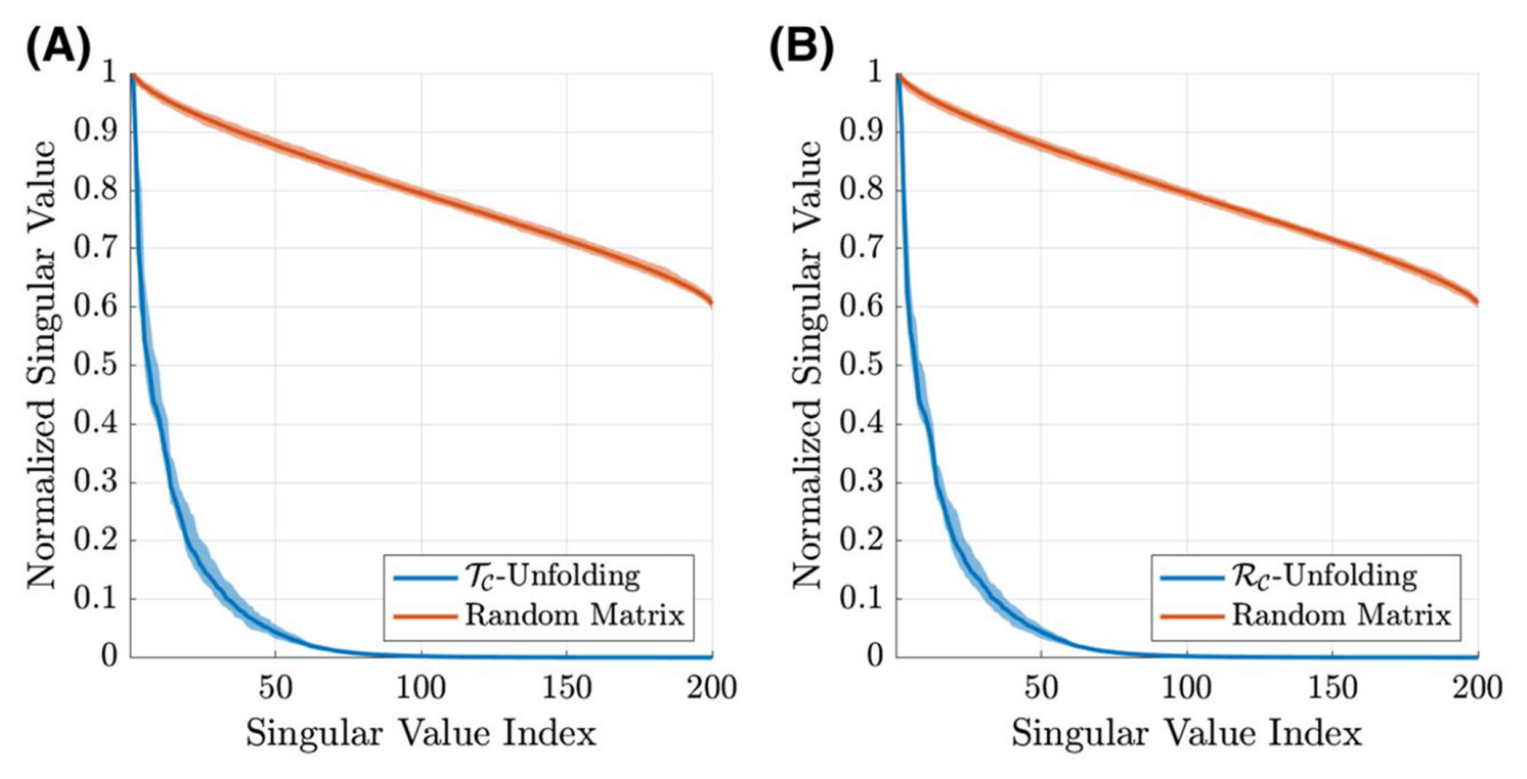
Singular value distributions for the transmit (*𝒯_𝒞_* A) and receive (*ℛ_𝒞_*, B) unfoldings of the synthetic data (blue). The shaded regions show the minimum and maximum range for the singular values across all slices in the dataset. In orange, singular value distributions for random matrices are shown for comparison

Transmit concatenation: We recognize here that the role to the transmit sensitivities is entirely analogous to that of the receive sensitivities, and that if *ℛ_𝒞_* should be well characterised with a low-dimensional subspace (ie, it has low rank), we should also be able to form a symmetrical unfolding:

(4) $$U_{1}:{\mathbb{C}}^{N_{1}\times N_{2}\times N_{R_{x}}\times N_{T_{x}}}\to {\mathbb{C}}^{\left(N_{1}\cdot N_{T_{x}}\right)\times \left(N_{2}\cdot N_{Rx}\right)},$$

such that the matrix *𝒯_𝒞_* = *U*_1_ *ℋ* (Figure 1E) also has low rank (Figure 2). All tensor unfoldings can be achieved through permutation and reshaping of the multi-dimensional array. However, we note that *𝒯_𝒞_* is not simply the transpose of *ℛ_𝒞_* because the block-Hankel front slices of the tensor are not changed when the receive and transmit dimensions are swapped.

One key idea that we explore here is not only the alternative choice of transmit and receive concatenations (compared to the PRIMO-style concatenation) but also the use of both representations and the simultaneous enforcement of low-rank structure in both *𝒯_𝒞_* and *ℛ_𝒞_* unfoldings. We propose to use this structure of the transmit and receive k-space to exploit all the correlations and redundancies in the multi-dimensional data by reconstructing undersampled k-space transmit and receive data through the use of low-rank constraints across *multiple* simultaneous unfoldings of the data tensor and by taking advantage of the symmetry between the transmit and receive dimensions.

### 2.2 Low-rank constraints

We can formulate the reconstruction problem as a convex problem using a sum-of-nuclear norms approach as follows:

(5) $$\min\frac{1}{2}\|M_{𝓏}-D{\|}_{F}^{2}+\lambda_{1}\|U_{1}T𝓏{\|}_{*}+\lambda_{2}U_{2}T𝓏{\|}_{*}$$

However, in this work we choose to employ an alternative non-convex formulation with strict rank constraints, which performs well in this context, and non-convex formulations have been used effectively in previous applications.^16,17^ It also has a more intuitive constraint parameterization (discrete rank thresholds rather than continuous regularization parameter *λ*):

(6) $$\begin{array}{l} \underset{𝓏}{\min}\frac{1}{2}\|M𝓏-D{\|}_{F}^{2}\\ \text{subject}\;\text{to:}\;rank\left(T_{𝒞}\right)=r_{1}\text{. }\\ \text{subject}\;\text{to:}\;rank\left({ℛ}_{𝒞}\right)=r_{2} \end{array}$$

Here, *M* is the k-space sampling mask (applied to *𝓏* as an element-wise Hadamard product); *𝓏* is the reconstructed k-space tensor; *𝒟* is the undersampled k-space data; *𝒯_𝒞_* = *U*_1_ *T𝓏* ; *ℛ_𝒞_* = *U*_2_ *T𝓏*; *r_i_* are the rank thresholds; and ∥·∥_*F*_ denotes the Frobenius norm. We will refer to this method for accelerated relative parallel transmit mapping as the transmit low rank (TxLR) method, which we can also reference using the constraint shorthand *𝒯_𝒞_* + *ℛ_𝒞_*.

Using the same reconstruction framework, with only changes to the number of constraints and the type of unfolding operator used, we can formulate a VC reconstruction that is the analogue of the virtual coil (VC) approach:

(7) $$\begin{array}{l} \underset{𝓏}{\min}\frac{1}{2}\|M𝓏-D{\|}_{F}^{2}\\ \text{subject}\;\text{to:}\;rank\left(V_{𝒞}\right)=r_{0} \end{array}$$

where *𝒱_𝒞_* = *U*_0_ *T𝓏*.

We will refer to the *ℛ_𝒞_*-constrained reconstruction as the PRIMO approach due to the correspondence between the transmit and receive concatenation order in *ℛ_𝒞_* and the relative transmit mapping calibration matrix defined by PRIMO. This is formulated as:

(8) $$\begin{array}{l} \underset{𝓏}{\min}\frac{1}{2}\|M𝓏-D{\|}_{F}^{2}\\ \text{subject}\;\text{to:}\;rank\left({ℛ}_{C}\right)=r_{2} \end{array}$$

where *ℛ_𝒞_* = *U*_2_ *T𝓏*. The difference between the proposed TxLR approach and the PRIMO approach is the additional simultaneously enforced *𝒯_𝒞_* constraint in the TxLR formulation.

## 3 Methods

### 3.1 Algorithm

The constrained optimisation problem in Equation (6) was solved using the alternating direction method of multipliers (ADMM)^26^ algorithm:

(9) $$\begin{array}{l} \text{ (i): }T_{c}^{n+1}={\text{Γ}}_{r_{1}}\left(U_{1}T{𝓏}^{n}-{𝓎}_{1}^{n}\right)\\ (ii):{ℛ}_{c}^{n+1}={\text{Γ}}_{r_{2}}\left(U_{2}Tx^{n}-{𝓎}_{2}^{n}\right)\\ (\text{iii }):{𝓏}^{n+1}=\underset{𝓏}{argmin}\frac{1}{2}\|M𝓏-D{\|}_{F}^{2}+\frac{\rho }{2}\|T_{C}^{n+1}-U_{1}T𝓏+{𝓎}_{1}^{n}{\|}_{F}^{2}+\frac{\rho }{2}\|{ℛ}_{C}^{n+1}-U_{2}T_{𝓏}+{𝓎}_{2}^{n}{\|}_{F}^{2}\\ (iv):{𝓎}_{1}^{n+1}={𝓎}_{1}^{n}+T_{C}^{n+1}-U_{1}T{𝓏}^{n+1}\\ (v):{𝓎}_{2}^{n+1}={𝓎}_{2}^{n}+{ℛ}_{C}^{n+1}-U_{2}T{𝓏}^{n+1} \end{array}$$

where superscript denotes the iteration number; *𝓎*_1_ and *𝓎*_2_ are auxiliary variables; and*ρ* is the ADMM penalty parameter. The final output *𝓏^n^* after *n* iterations is a k-space by transmit and receive tensor with the same dimensions as the undersampled *𝒟*. Γ_*r*_ is a singular value hard-thresholding operator that performs an SVD and discards all singular values with index *i* > *r* when ordered in decreasing magnitude. The algorithm is initialised with all variables, including *𝓏* ^0^, ${𝓎}_{1}^{0}$, and ${𝓎}_{2}^{0}$ set to 0 (a matrix or array of zeros).

All reconstructions were implemented using MatLab R2019b (Mathworks, Natick, MA). The VC (Equation (7)) and PRIMO (Equation (8)) reconstructions were performed analogously by omitting steps (ii) and (v), adjusting the second term in (iii) accordingly, and using the appropriate unfolding operator *U*_0_ or *U*_2_. In practice, the algorithm also uses a varying penalty parameter *ρ*, with *ρ* ^0^ = 10^−6^ and scaling factor *τ* = 1.1, and takes advantage of over-relaxation with parameter *α* = 1.5 to improve convergence^26^ (a complete description of the algorithm, including these parameters, is provided in the Appendix).

Reconstructions were run to a fixed number of iterations or using a chi-square heuristic based on receive array noise characteristics. The chi-square heuristic checks, at every iteration, the condition:

(10) $$\overset{N_{Rx}}{\underset{i=1}{\sum^{\text{​}}}}\frac{\|M{\overset{}{𝓏}}_{i}-D_{i}{\|}_{F}^{2}}{\sigma_{i}^{2}}/v>1\text{​}$$

where *σ_i_* is the noise variance from the *i^th^* receive channel (obtained from a separately acquired noise reference scan), and *υ* is the total number of sampled data points. This expression is the well-known chi-square goodness-of-fit test, and we use it here to provide a parameter-free stopping criterion.

### 3.2 Data

In-silico data were simulated in Sim4Life 3.4 (ZMT, Zurich, Switzerland) using an 8-channel transmit/receive dipole array^27^ at the 7 Tesla frequency of 298 MHz, centred over the heart of Duke^28^ (Virtual Population, iTIS Foundation, Zurich, Switzerland). The maximum resolution around the conductors was set to 0.5 mm, 1.0 mm, or 2.0 mm as required to capture the geometry. A synthetic proton density image was generated by setting the proton density equal to the tissue density, with tissue densities greater than 1200 kg/m^3^ (bone) or less than 400 kg/m^3^ (lung) set to 80 (a/u) to make them resemble an MR image. The FOV was 278 × 356 × 248 mm^3^ (AP/LR/HF), and data were re-sampled onto a uniform grid of 2 × 2 × 2 mm^3^. This data were Fourier-transformed to form a k-space dataset, and 48 central slices located in the body were used for reconstruction simulations.

In vivo data were acquired on a 7 Tesla Magnetom (Siemens, Erlangen, Germany), and all volunteers provided written informed consent and were scanned in accordance with local ethics. Data were acquired using a spoiled low flip angle gradient echo acquisition, which transmitted on 1 channel at a time while receiving on all channels (see Supporting Information Table S1 for details of the protocols used). In the brain, data were acquired in a sagittal orientation using an 8-channel transmit, 32-channel receive coil (Nova Medical, Wilmington, DE), and a 3D acquisition. In the body, an 8-channel transmit/receive dipole array was used,^27^ and 2D images were acquired in a horizontal long axis orientation. Receive array noise measurements were also collected to generate noise covariance matrices for the chi-square convergence heuristic.

All simulations using the synthetic data were performed with added channel-independent noise at peak SNR (defined as the maximum signal divided by the noise SD) of 60 dB in k-space unless otherwise noted. Retrospective undersampling using a uniform density pseudo-random Poisson disc distribution was employed to assess reconstruction performance at acceleration factors ranging from *R* = 2 to *R* = 12.

### 3.3 Reconstruction parameters

Reconstructions were performed using a [5,5] kernel, and rank thresholds were set to 50 unless otherwise stated. The number of iterations was set to 50 for the TxLR reconstructions, and 100 for the VC and PRIMO methods in all synthetic data reconstructions, except in the cases where the chi-square heuristic was evaluated. See Supporting Information Figure S1 for validation of these parameter choices. The chi-square stopping criteria was used in all reconstructions of the in vivo body and brain data.

In all cases, the k-space data were cropped to a matrix size of 24 × 24, except where the impact of k-space matrix size was explicitly evaluated, which examined crop sizes of 18 × 18, 24 × 24, 36 × 36, and 48 × 48. For the 3D datasets (synthetic body, and in vivo brain), reconstruction was performed slice by slice on hybrid space kx-ky-z data.

Reconstruction fidelity was evaluated using a normalized RMSE metric:

(11) $$RMSE=\frac{\|\hat{𝓏}-𝓏{\|}_{F}}{\|𝓏{\|}_{F}}$$

where $\hat{𝓏}$ is the estimated k-space tensor, and *𝓏* is the ground truth. Empirically, we found that an RMSE < 0.1 generated small errors in relative transmit sensitivity maps (<4%, everywhere in the image). Relative transmit sensitivity maps were estimated using the PRIMO extension to ESPIRiT after under-sampled tensor reconstruction.

## 4 Results

**Figure 3:**
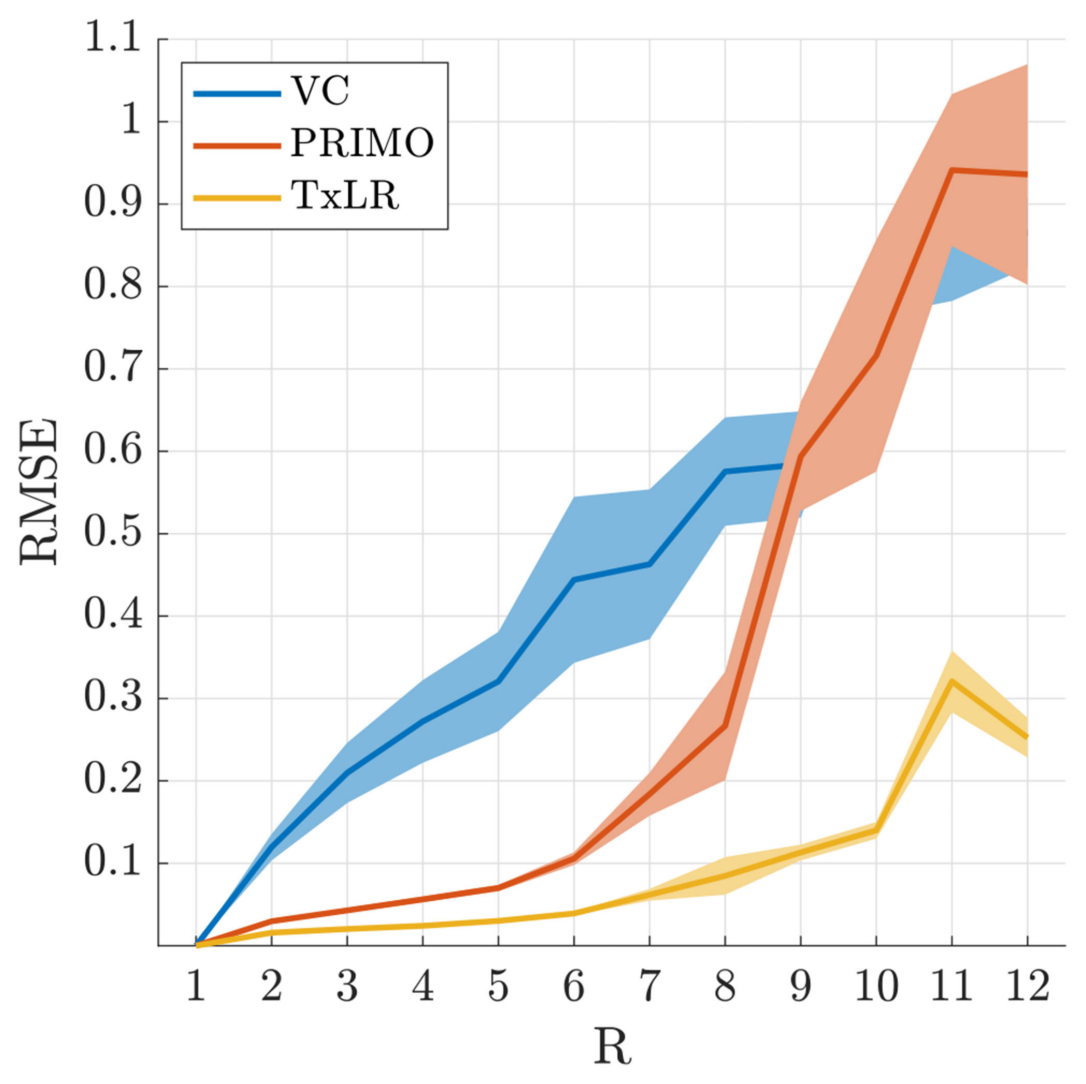
RMSE for the VC (blue), PRIMO (orange), and TxLR (yellow) methods evaluated in the synthetic body dataset at different acceleration factors. Shaded regions indicate the SD of the RMSE across 48 slices

Reconstruction performance on the synthetic body dataset is shown in Figure 3 for acceleration factors ranging from *R* = 2 to *R* = 12 for the VC approach (*𝒱_𝒞_* constraint), PRIMO approach (*ℛ_𝒞_* constraint), and the proposed TxLR approach (*𝒯_𝒞_* + *ℛ_𝒞_* constraints). Mean RMSEs for all methods are shown, along with their SDs across all 48 reconstructed slices. Whereas the VC reconstruction reaches RMSE > 0.1 at *R* = 2, the PRIMO reconstruction achieves *R* = 6 undersampling before reaching that error threshold. In comparison, the TxLR reconstruction manages to reach acceleration factors of *R* = 8 before showing the same error levels. Furthermore, both the VC and PRIMO reconstructions suffer from very poor reconstruction fidelity as acceleration factors increase, whereas the proposed TxLR approach shows a much lower rate of error increase with increasing *R*.

**Figure 4:**
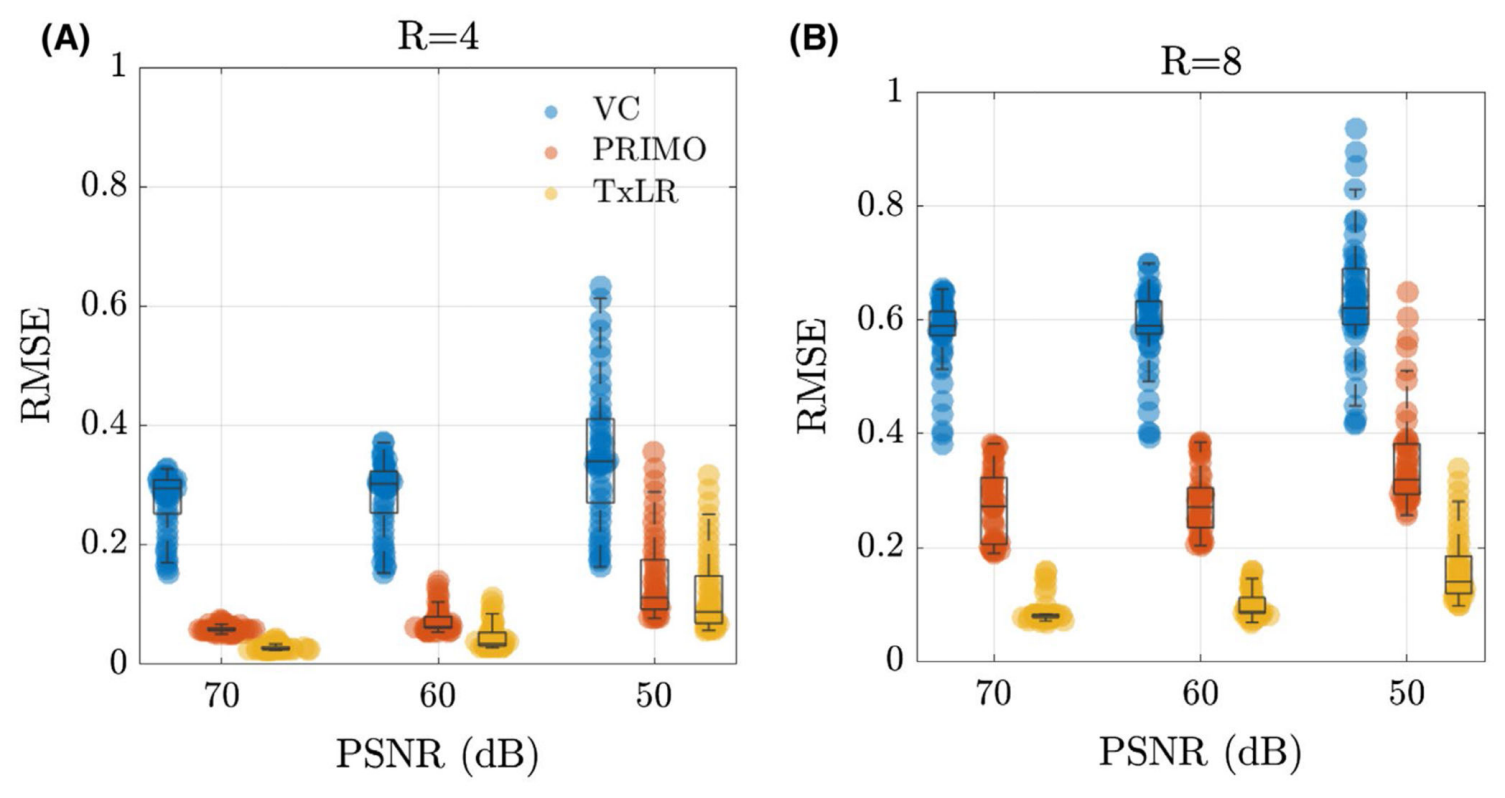
RMSE in the synthetic data at varying PSNR and acceleration factors. Each VC (blue), PRIMO (orange), and TxLR (yellow) scatter point represents the RSME for a single slice. Acceleration factors (A) R = 4, and (B) R = 8 are shown. PSNR, peak SNR

Reconstructions in the synthetic dataset were assessed at varying noise levels, with peak SNR ranging from 50 dB to 70 dB across acceleration factors of *R* = 4 and *R* = 8. Figure 4 plots the aggregated RMSE from all 48 slices, which shows the expected increase in RMSE with decreasing peak SNR and increasing acceleration factors across all methods. In all cases, the proposed TxLR reconstructions produce the lowest RMSE, although at the lower acceleration factor PRIMO performs nearly as well.

**Figure 5:**
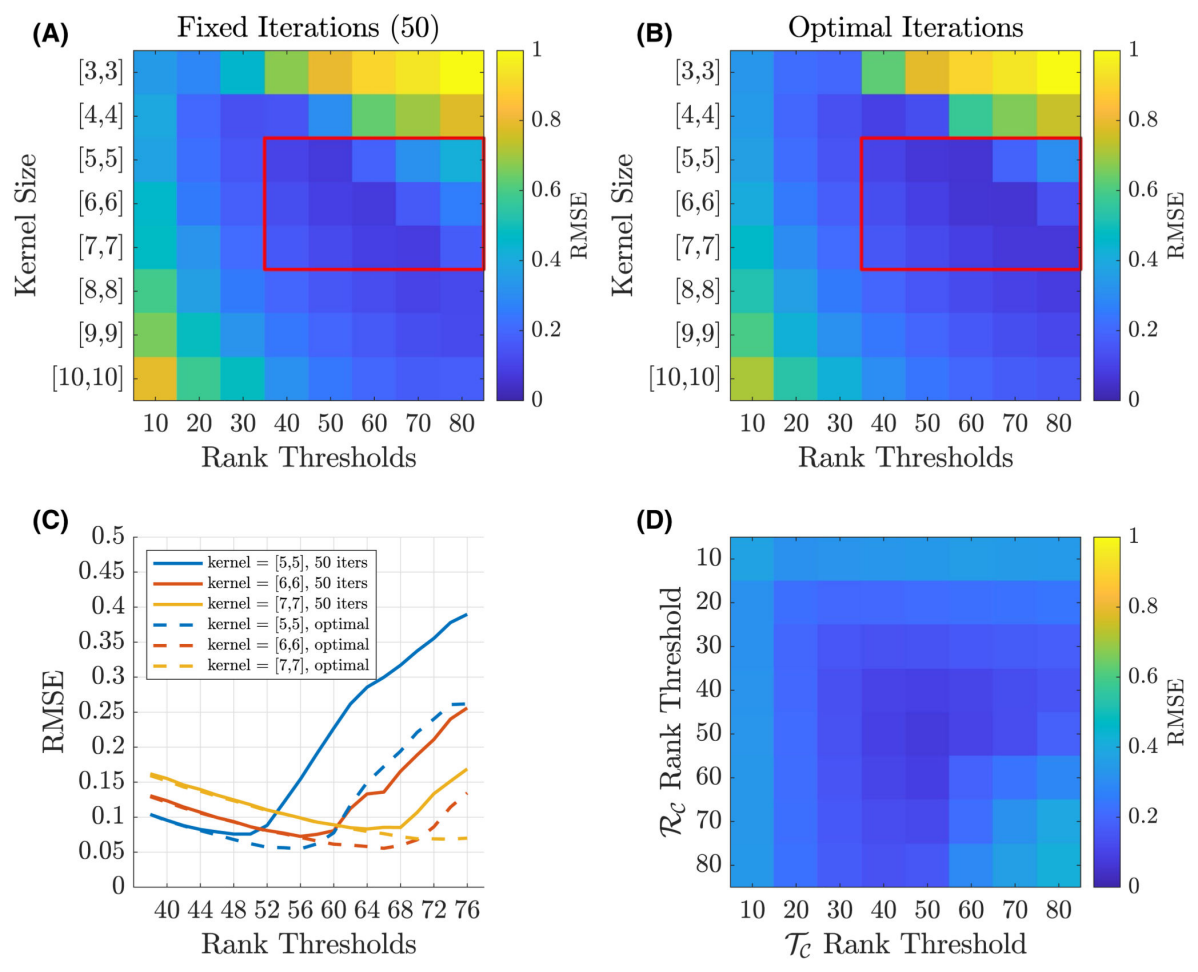
Exploring the impact of various parameter choices on the TxLR approach. (A) RMSE at different kernel sizes and rank thresholds using a fixed 50 iteration reconstruction; (B) similar to (A), but with a retrospectively selected number of iterations that produces the smallest RMSE; (C) line plot of the parameter space outlined in the red boxes of (A,B); (D) the result of varying the *𝒯_𝒞_* and *ℛ_𝒞_* constraints independently

In Figure 5, the impact of varying iterations, kernel size, and rank thresholds on the proposed reconstruction scheme was evaluated on a central slice of the synthetic dataset at *R* = 8. Figure 5A,B show the reconstructed RMSE across square kernels ranging from [3,3] to [10,10] and rank thresholds of 10 to 80 for both the *𝒯_𝒞_* and *ℛ_𝒞_* constraints. In Figure 5A, a fixed iteration count of 50 was used, whereas in Figure 5B the “optimal” number of iterations was used based on retrospectively choosing the number of iterations that minimized RMSE. In both cases, the minima can be found within the parameter subset highlighted by the red box, for which Figure 5C provides a more detailed comparison of RMSE performance. This highlights that a rank constraint of 50 in a fixed 50 iteration reconstruction produces near-optimal results in this data. Figure 5D shows the impact of varying the rank constraints of the *𝒯_𝒞_* and *ℛ_𝒞_* terms independently, indicating that RMSE is minimized when both rank constraints are the same and equal to 50 in this data. An evaluation of reconstruction performance for different matrix sizes and acceleration factors can be found in Supporting Information Figure S2.

**Figure 6:**
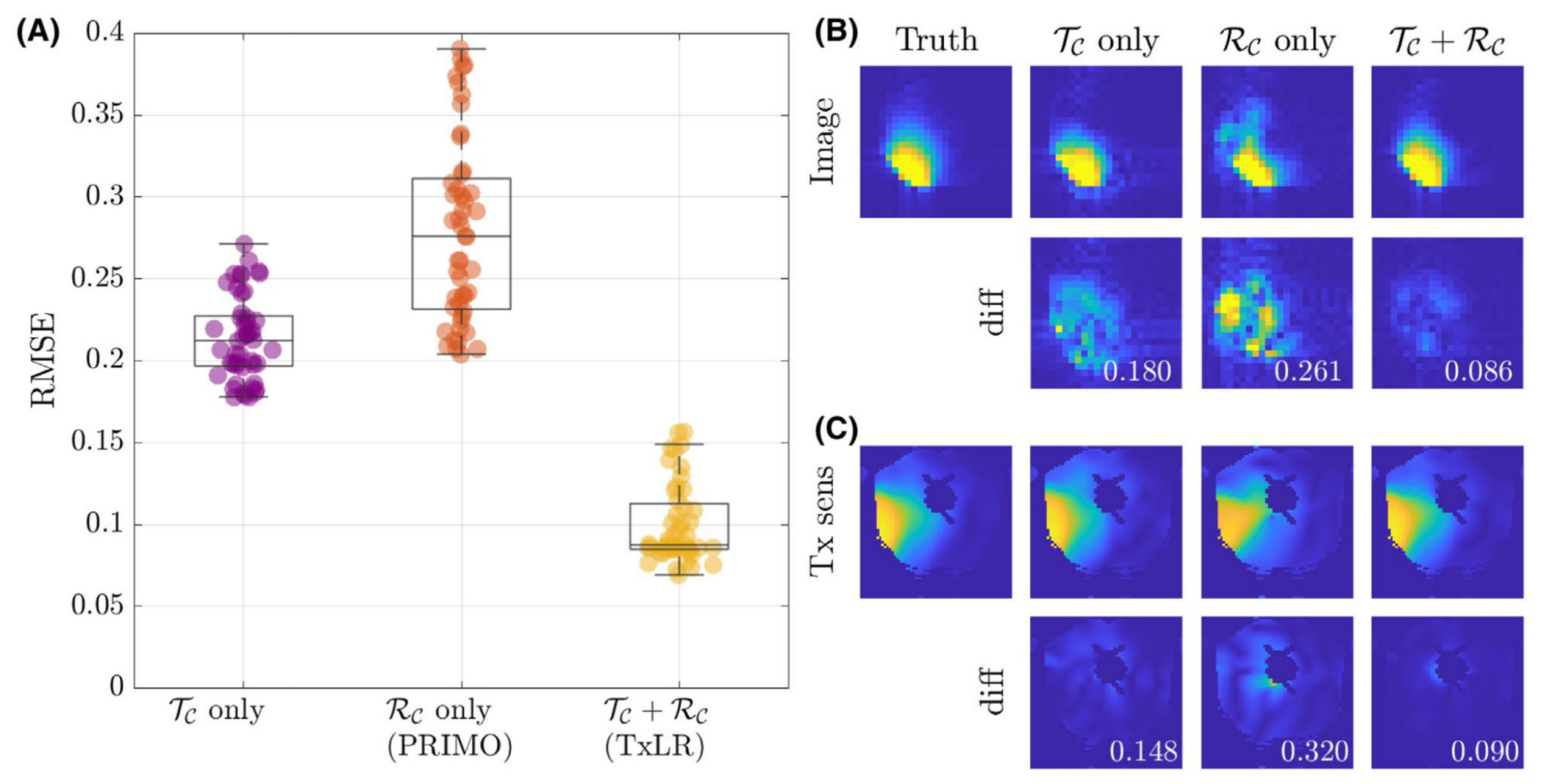
(A) RMSE in the synthetic data at R = 8, resulting from reconstructions using the transmit and receive (*𝒯_𝒞_*) constraint (purple), receive (*ℛ_𝒞_*) constraint (orange, PRIMO), and simultaneous transmit and receive (*𝒯_𝒞_* + *ℛ_𝒞_*) constraints (yellow, TxLR), with each marker corresponding to a single slice. (B) Reconstructed images corresponding to a single transmit and receive pair (transmit channel 4, receive channel 3) and (C) transmit field maps (transmit channel 4), at slice z = 24. The number inset in the difference images corresponds to RMSE

To further assess the value of the proposed approach, Figure 6 shows a comparison of using the *𝒯_𝒞_* or *ℛ_𝒞_* constraints individually, or simultaneously as proposed, evaluated again in the synthetic body dataset at *R* = 8. In Figure 6A, RMSE is shown to be similar for the *𝒯_𝒞_*- or *ℛ_𝒞_*-only reconstructions (where *ℛ_𝒞_* only is the same as the PRIMO approach). Whereas in this case, vertical concatenation of the transmit dimension (to form the *𝒯_𝒞_* matrix) slightly outperforms the *ℛ_𝒞_*-based PRIMO approach (vertically concatenating the receive dimension), the difference is relatively small. However, reconstruction fidelity is significantly better for the TxLR approach, which uses the simultaneous *𝒯_𝒞_* + *ℛ_𝒞_* constraints.

In Figure 6B, the top row shows a representative reconstructed image for all 3 approaches compared to the ground truth. The second row shows difference images and RMSE for that image, which is lowest in the *𝒯_𝒞_* + *ℛ_𝒞_* case. Figure 6C shows an estimated relative transmit sensitivity map and difference from ground truth. In both, visual qualitative inspection and RMSE indicate that the proposed *𝒯_𝒞_* + *ℛ_𝒞_* (TxLR) reconstruction produces the smallest errors.

An evaluation of the chi-square stopping heuristic is shown in Supporting Information Figure S3. This shows that both the choice to cut off iterations at 50 and the chi-square heuristic produced reconstructions close to optimal, although the heuristic tended to slightly overestimate the number of required iterations.

**Figure 7:**
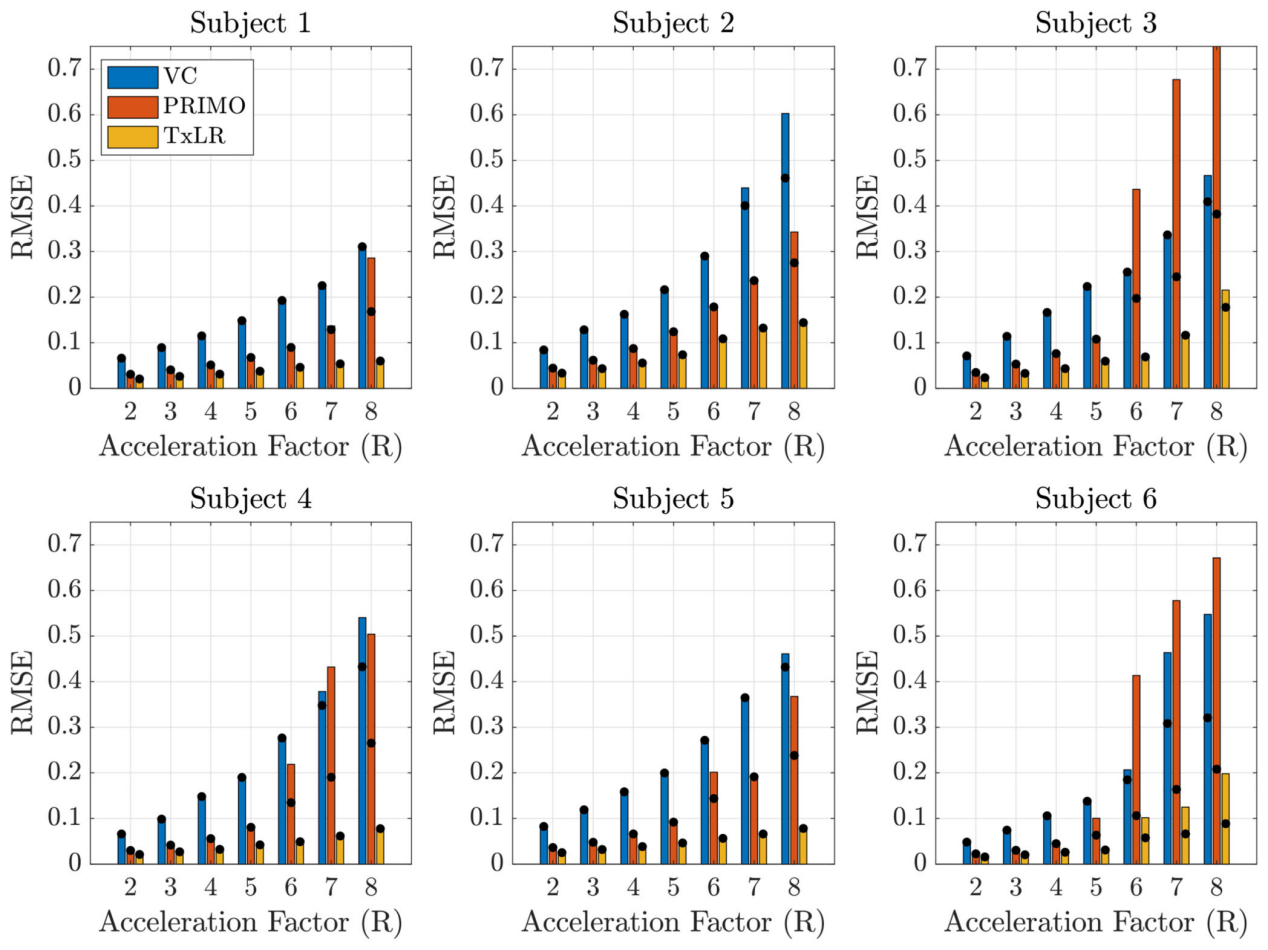
Results from all 6 subjects for the in vivo body dataset at acceleration factors of R = 2-8. Bars denote the RMSE at the fixed rank threshold of 50, and black dots denote the RMSE assuming an optimal choice of rank threshold

**Figure 8:**
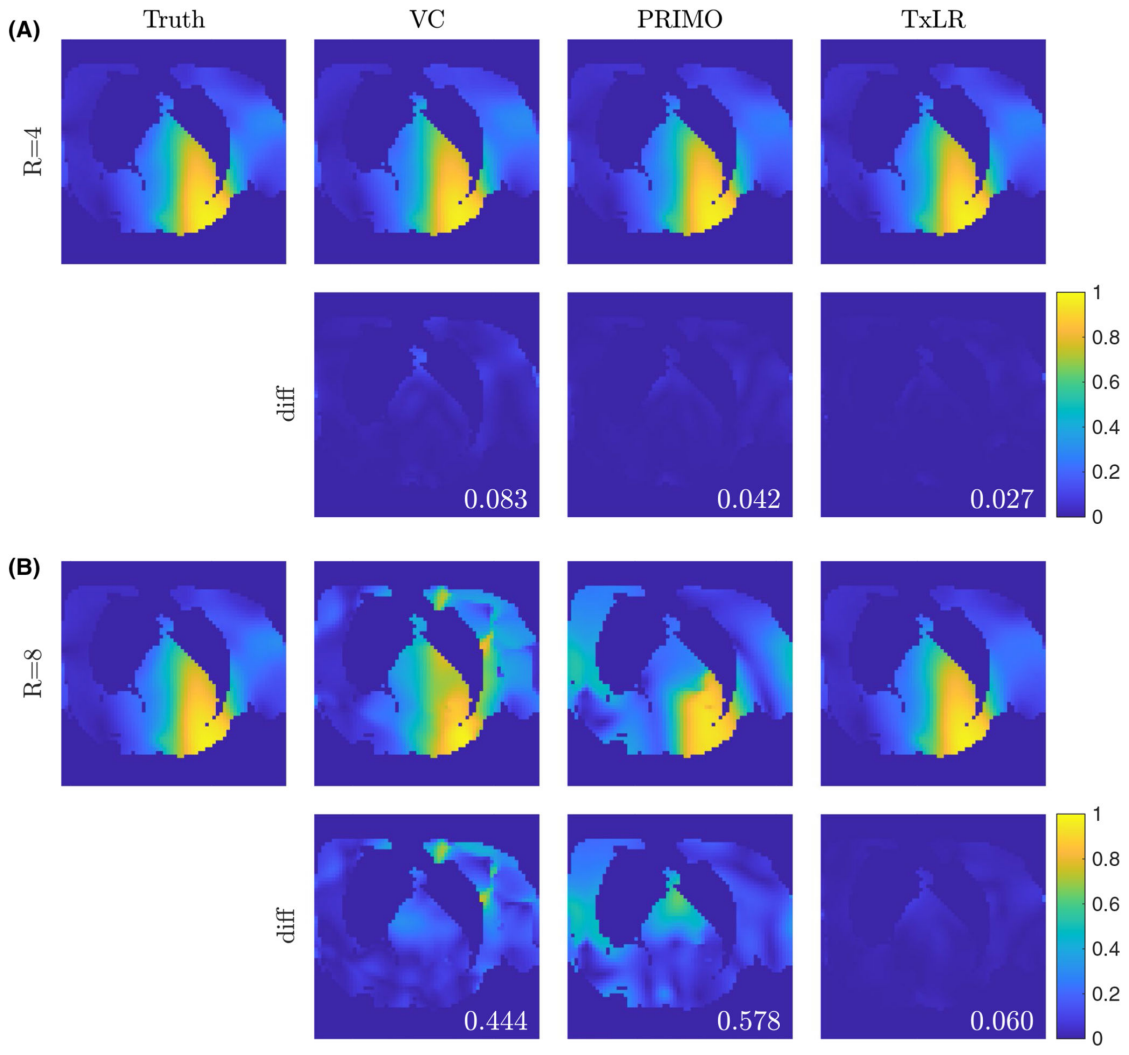
Estimated transmit sensitivity maps and the magnitude of the complex differences to the ground truth for subject 4, transmit channel 3, at (A) R = 4 and (B) R = 8. The values in each difference map correspond to the sensitivity map complex RMSE compared to ground truth

Figures 7 and 8 show the reconstruction results for the in vivo 2D body datasets. In Figure 7, RMSE results for all subjects can be seen. The bar values represent the RMSE at a rank constraint of 50 compared to an *optimal* rank constraint between 5 and 50, as indicated by the black dot. For the best performing subject, RMSE remained below 0.1 for the proposed TxLR method even at R = 8 acceleration, whereas in the worst performing subject RMSE reached as high as 0.2 at R = 8. However, in all subjects and all acceleration factors, the TxLR reconstructions produced lower RMSE than the VC or PRIMO methods, although the differences between TxLR and PRIMO were relatively small for R ≤ 4. This relative performance held for both the fixed rank constraint of 50 and the retrospectively chosen *optimal* rank thresholds. In subjects 1 to 5, the rank constraint of 50 was optimal or close to optimal for the VC and TxLR reconstructions, whereas the PRIMO data benefitted from reduced rank thresholds. In subject 6, lower rank thresholds produced considerably lower RMSE for all methods, which may reflect orientation differences in that dataset compared to the others.

In Figure 8, we see a set of relative transmit sensitivity maps and their difference images at R = 4 and R = 8 for a representative subject using the rank 50 reconstructions. Although the differences between methods are small at R = 4, consistent with the results in the synthetic data, at R = 8 only the TxLR method produces a Tx-sensitivity map that resembles the ground truth, with comparable error to the R = 4 reconstruction.

**Figure 9:**
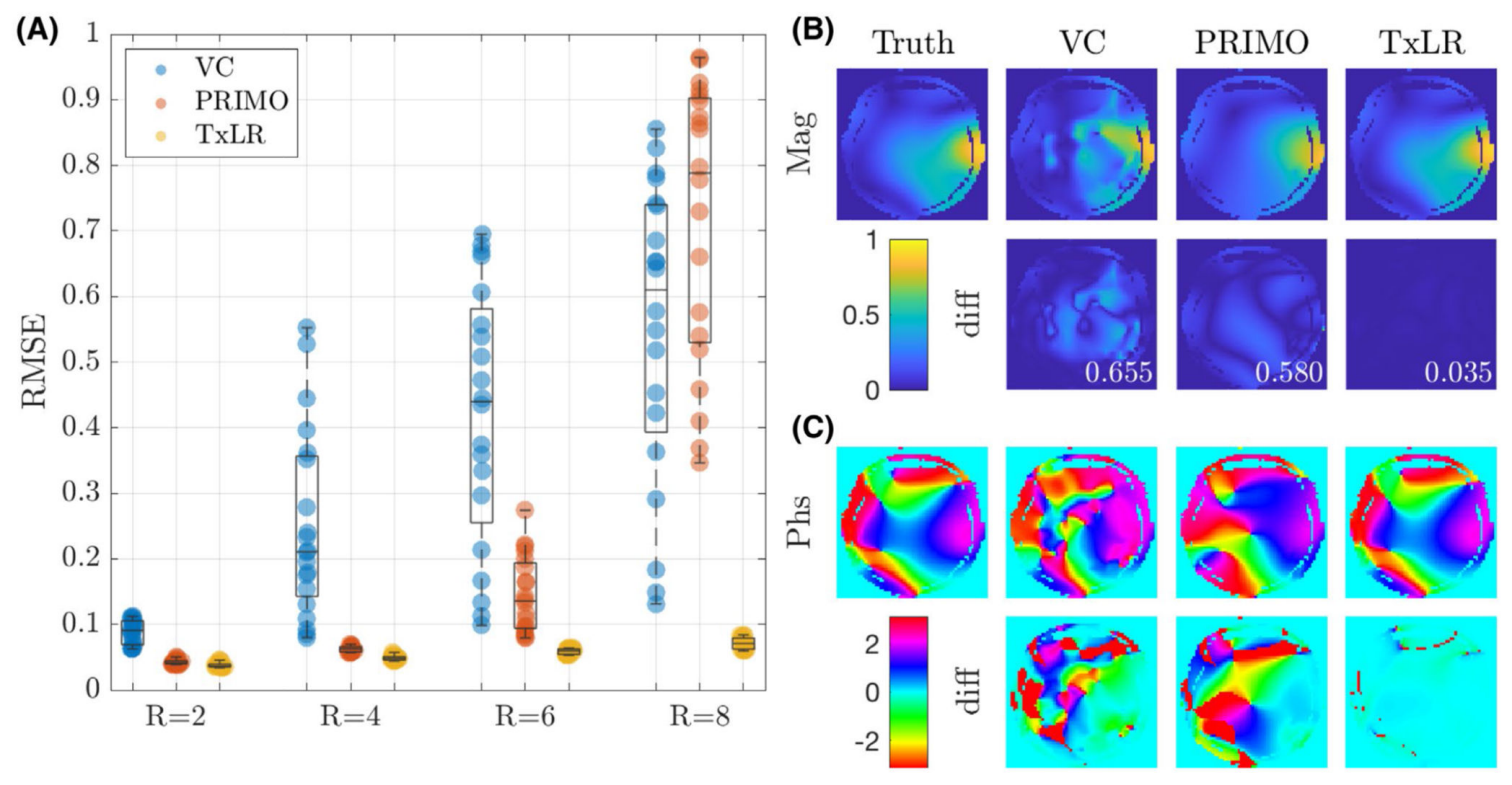
Results from the in vivo brain dataset. (A) RMSE at acceleration factors R = 2, 4, 6, and 8, for the VC method (blue); PRIMO (orange); and TxLR (yellow), with markers representing the RMSE for each of the 36 slices. (B,C) Representative (transmit channel 4) transmit sensitivity maps showing (B) magnitude maps and magnitude differences, and (C) phase maps and phase difference images. The values inset on the difference maps in (B) correspond to the complex RMSE compared to ground truth

Figure 9 shows the reconstruction results for the 3D in vivo brain dataset. In Figure 9A, the RMSE across 36 central slices is shown, with the TxLR data showing a dramatic improvement in RMSE compared to the VC or PRIMO reconstructions at higher acceleration factors (R = 6, 8), which could reflect better use of the increased redundancy provided by the 32-channel receive array (compared to the 8-channel receive used in the synthetic and experimental body data). Even at R = 8, all brain slices were below RMSE of 0.1 (min = 0.060, max = 0.084). In contrast, the VC results show significantly increased RMSE beyond R = 2 and PRIMO beyond R = 4.

Figure 9B,C show the magnitude and phase of relative transmit sensitivity maps and their difference images from the ground truth for transmit channel 4 for the R = 8 data. The maps derived from the TxLR reconstructions show clear benefit over the other approaches and are virtually indistinguishable from the ground truth in both magnitude and phase.

Reconstruction times (measured using an Intel Core i9, 8-core 2.3 GHz processor, Santa Clara, CA, USA) in the body dataset were 0.18 s/iteration/slice (VC), 0.16 s/iteration/slice (PRIMO), and 0.20 s/iteration/slice (TxLR) for overall reconstruction times of approximately 10 to 20 s/slice. In the brain dataset, with 32 receive channels instead of 8, reconstruction times were 0.7 s/iteration/slice (VC, PRIMO) and 1.0 s/iteration/slice (TxLR) for total reconstruction times of approximately 50 to 70 s/slice.

## 5 Discussion

This work investigated the use of Hankel-structured low rank tensor completion to reconstruct undersampled parallel transmit field maps. This leverages the fact that both transmit and receive sensitivity maps possess low rank properties when represented as a Hankel-structured matrix. Redundancy in a dataset in which every transmit sensitivity is modulated by every receive sensitivity is intuitively evident, as data with *N_Tx_* transmit channel and *N_Rx_* receive channels results in *N_Tx_* · *N_Rx_* images, but only at most *N_Tx_* + *N_Rx_* of them are linearly independent.

We investigated 3 different approaches for simultaneously reconstructing transmit and receive sensitivity maps in a single rank-constrained reconstruction. We formulated these all in a comprehensive multi-dimensional tensor model (kx by ky by *N_Tx_* by *N_Rx_*), with the 3 approaches differing in how the low-rank constraints are imposed onto the tensor (via different matricizations or tensor-unfoldings).

The first method investigated (VC) is similar to the virtual coils approach in time interleaved acquisition of modes,^25^ where the transmit and receive sensitivity combinations are used as virtual coils with the number of virtual coils equal to *N_Tx_* · *N_Rx_*. The low-rank constraint was imposed on a tall matrix formed by the vertical concatenation of the block-Hankel matrix from each “virtual coil.” However, this reconstruction rapidly deteriorated as the acceleration factor increased. This is likely because the matrix dimensionality is not well suited to the low rank constraint; the matrix becomes significantly longer than it is wide (ie, there are more coils than elements in kernel). This is also reflected in the SNR performance, where the virtual coils approach shows significantly worse RMSE with lower SNR.

The second method (PRIMO) uses the same transmit and receive channel concatenation scheme as PRIMO,^13^ with the low-rank constraint enforced on a matrix formed by vertically concatenating the block-Hankel matrices from each receive channel and horizontally concatenating each transmit channel. This results in a matrix with similar height and width and a more effective low-rank representation. This approach performed significantly better than the VC method at lower acceleration factors (R ≤ 4) but exhibited similar or worse performance at high acceleration factors.

The third proposed transmit low-rank method (TxLR) makes use of the symmetry in the transmit and receive array data by employing simultaneous low-rank constraints on 2 different unfoldings of the tensor data. One constraint is identical to the PRIMO constraint, whereas the second constraint enforces low-rankness on a matrix formed by horizontal receive concatenation and vertical transmit concatenation. The use of both these constraints is based on the recognition of the interchangeable nature of the transmit and receive sensitivities, where we demonstrated that each of these unfoldings individually have low-rank structure and perform similarly using an 8-channel transmit and 8-channel receive system. However, the 2 unfoldings capture *different* low-dimensional features in the tensor data and are not trivially related through a transpose operation; therefore, leveraging the low-rankness of both of the unfoldings simultaneously constitutes a more powerful constraint, leading to dramatically improved reconstruction fidelity as assessed by RMSE.

In all cases, the TxLR approach clearly performed better than the other methods, particularly at higher acceleration factors. At lower acceleration factors, RMSEs were similar to those produced by the PRIMO method; however, dramatic differences were observed at R = 6 and R = 8. TxLR reconstructions in an 8-channel transmit system achieved acceleration factors of R = 8 with low RMSE in the body (0.1 in the best performing dataset, to 0.2 in the worst case) and the brain (RMSE < 0.1 for all slices). These results indicate that relative transmit calibration can be achieved in the same amount of time or faster than B_0_ calibration. The reconstruction performance in the brain data suggested that TxLR benefits from additional receive channels (32 in the brain compared to 8 in the body), although this is difficult to determine with only a single brain dataset.

Reconstruction hyperparameters (kernel size = [5,5], rank threshold = 50) were selected based on the synthetic dataset. These hyperparameters were able to robustly produce high-fidelity reconstructions in the in vivo body and brain datasets without any tuning to the specific coil geometries or organ. However, the parameters determined here are not universal recommendations. Different resolutions, matrix sizes, SNR regimes, and coil configurations may require different kernel sizes and rank thresholds; and context-specific parameter tuning will likely be required for optimized reconstructions. In the body dataset, for example, we did show that the chosen rank threshold of 50 was not always optimal, suggesting that data-specific hyperparameter tuning could lead to further reductions in RMSE. This subject-to-subject variation is likely driven by the slice orientation and not the FOV, as evidenced in Figure 7, where the rank threshold of 50 remained optimal despite a change in the FOV between subjects 1 and 2. In addition, because of the nonconvex problem formulation, stopping criteria for the iterative reconstruction can be important to avoid diverging from a local optimum. Here we show that, with knowledge of the channel-wise receiver noise characteristics, using a chi-square stopping heuristic resulted in near-optimal reconstruction performance.

Accelerations of eightfold (matching the number of transmit states) worked best for an image matrix of 36 × 36; however, a 24 × 24 matrix is sufficient to capture the dominant transmit modes in the heart (see Supporting Information Figures S4 and S5), where a sixfold acceleration is reliable for the heart data. A 3D relative transmit mapping acquisition on an 8-channel system requires 4608 (24 × 24 × 8) lines of k-space data taking 16.1 s (TR = 3.5 ms) or 41 heartbeats (cardiac window of 400 ms). An acceleration factor of 6 brings this down to 2.1 s or only 7 heartbeats for the whole torso. This becomes more significant when increasing the transmit channel count; for example, mapping a 32-channel transmit system^29^ would take 65 s (24 × 24 × 32 × 3.5 ms), or 161 heartbeats; accelerating by a factor of 6 would bring this down to only 11 s, or 27 heartbeats.

The results presented here, although showing a clear benefit of the proposed approach for accelerated parallel transmit mapping, have several limitations that could be addressed in further development of the method. For example, the current study only examined uniform density Poisson disc k-space sampling, given the relatively small, central k-space sampling windows used for sensitivity mapping. Exploration of variable density sampling schemes would be a natural extension of this work, and initial simulations suggest that both convergence speed and lower RMSE could improve with such schemes (see Supporting Information Figure S6). Furthermore, the proposed method did not explore the use of phase constraints, like those used in Low-rank modeling of local k-space neighborhoods with parallel imaging (P-LORAKS),^15^ which could further improve performance by exploiting low-rank properties of conjugate symmetric k-space locations under smooth phase assumptions.

While this paper was under review, a method for multi-contrast Hankel tensor completion was published, which also leveraged a low-rank Hankel-structed tensor model for undersampled image recovery.^30^ In agreement with our work, they found improved reconstruction fidelity when multiple acquisitions are jointly reconstructed in a low-rank tensor framework by enforcing low-rankness on different tensor unfoldings simultaneously. However, there are several differences in the 2 approaches. Whereas the unfoldings used in the multi-contrast Hankel tensor completion approach are analogous to the *𝒱_𝒞_* and *ℛ_𝒞_* unfoldings, our approach did not use the virtual coil *𝒱_𝒞_* unfolding; instead, leveraging unfoldings reflected the symmetry in the receive and transmit channel dimensions. Furthermore, the multi-contrast Hankel tensor completion work used an alternating projection-onto-sets algorithm for tensor recovery, whereas in this work the tensor recovery problem was solved using the ADMM algorithm.

The TxLR approach could also be applied to absolute B1+ mapping of pTx systems by reconstructing multiple transmit configurations together (see supporting informatin “Simulation of accelerated absolute B1+ mapping” and Figure S7). The 3D DREAM method proposed by Ehses^31^ uses GRAPPA with a receive sensitivity pre-scan to accelerate the acquisition and reduce the echo train length. TxLR acceleration could be used to apply this method to parallel transmit mapping, removing the need for a pre-scan and enabling higher acceleration rates. Alternatively, it could be used to accelerate methods such as B1 time interleaved acquisition of modes (B_1_TIAMO)^9^ or interferometry methods^32^ by accelerating the relative mapping part of the acquisition and applying these maps to reconstruct undersampled absolute maps.

Using TxLR in practice may be limited by the reconstruction time of the current implementation; although not prohibitively long, care must ensure that further data can be acquired while reconstruction is in process. A rapid acquisition time will improve the robustness of the acquisition by reducing its susceptibility to motion and may further enable the characterisation of motion-induced field changes.

## 6 Conclusion

Calibrationless low-rank image reconstruction can be used to reconstruct transmit array sensitivity maps in highly accelerated conditions. Transmit low rank method harness redundancies in both transmit and receive coil profiles to enable reliable acceleration of transmit maps without the need for a calibration scan. The method enables acceleration factors of 8, equal to the number of transmit coils.

## Supplementary Material

Supporting Info

Appendix

## Data Availability

All data are provided for reproducibility at the following location: https://doi.org/10.5287/bodleian:YQpGNevaa. Reconstruction code and figure-generating code are also made fully available at https://github.com/mchiew/txlr_paper.
