# Supplementary material for: Accelerated calibrationless parallel transmit mapping using joint transmit and receive low-rank tensor completion
Source: Magn Reson Med. Author manuscript; Available in PMC 2021 Nov 1. (PMC7611890; doi:10.1002/mrm.28880)
Supplement: Supporting Info [file EMS136202-supplement-Supporting_Info.docx]

# Supporting Information

Table S1: Relative transmit mapping acquisition protocols, all data were acquired fully sampled and k-space cropped to 24x24 unless otherwise stated.

|  | Brain – 3D | Body (1) – 2D | Body (2) – 2D | Body (3-6) – 2D |
| --- | --- | --- | --- | --- |
| FOV (mm) | 220 x 220 x 163 | 400 x 400 x 8 | 300 x 300 x 10 | 360 x 360 x 8 |
| Matrix | 128 x 128 x 96 | 144 x 129 | 144 x 144 | 128 x 128 |
| Flip angle / reference voltage | 3° / 66 V | 4° / 200 V | 4° / 200 V | 7° /200 V |
| Bandwidth (Hz/px) | 662 Hz/px | 694 Hz/px | 694 Hz/px | 797 Hz/px |
| TR (ms) | 6.0 ms | 3.6 ms | 3.5 ms | 3.2 ms |
| TE (ms) | 2.04 | 1.45 ms | 1.53 ms | 1.04 ms |
| Duration (s) | 688 s | 8 heart beats | | |


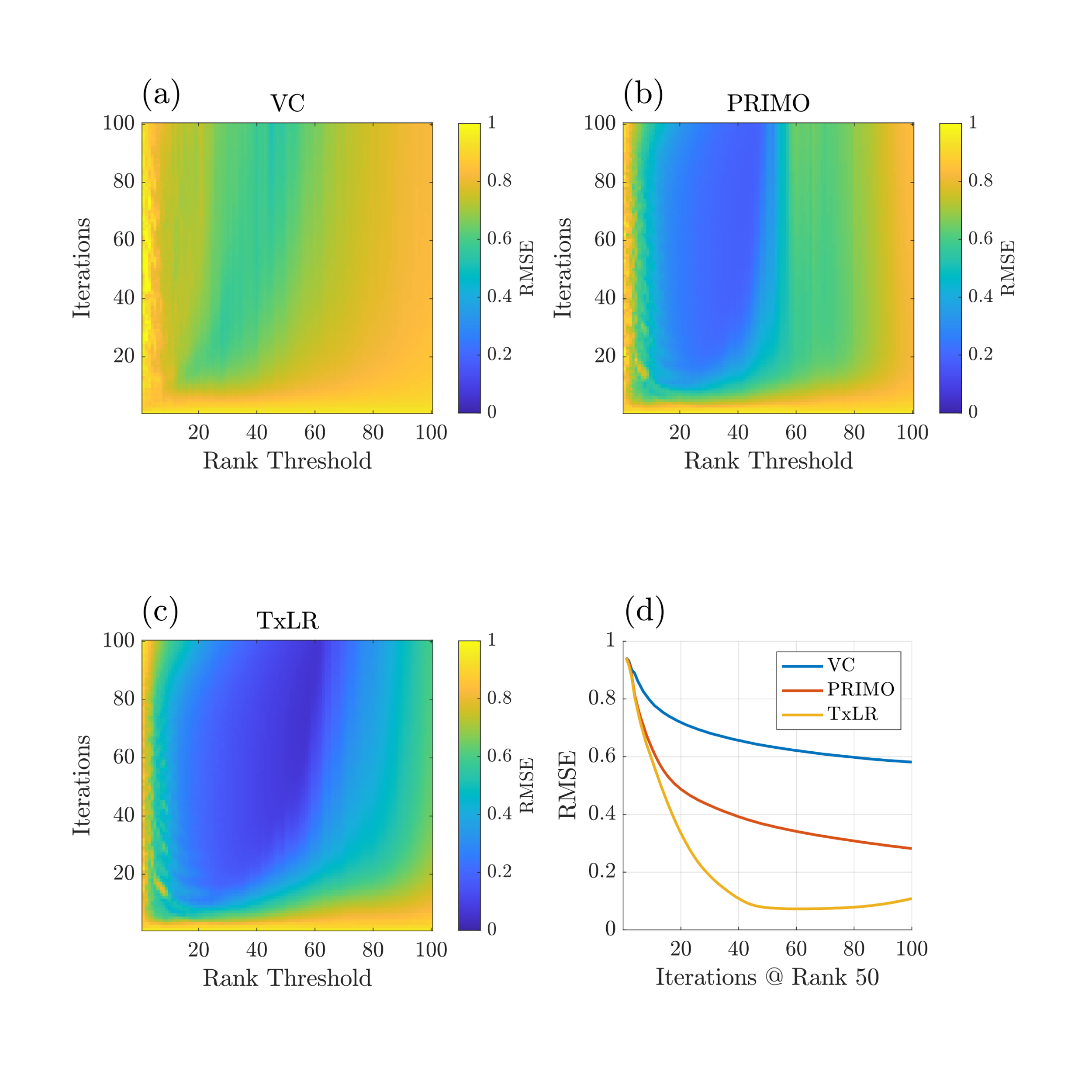


*Figure S1- RMSE for different iteration counts and different rank constraints. (a) VC method, (b) PRIMO, and (c) TxLR. (d) Plot of the RMSE vs iteration at rank 50.*

Figure S2 justifies the parameter choices made for the various reconstructions. All methods showed optimal RMSE around a rank threshold of 50. For the single constraint methods VC and PRIMO, optimality was achieved at the maximum evaluated iteration number of 100, whereas for the double constrained TxLR, optimality was achieved around iteration 50.


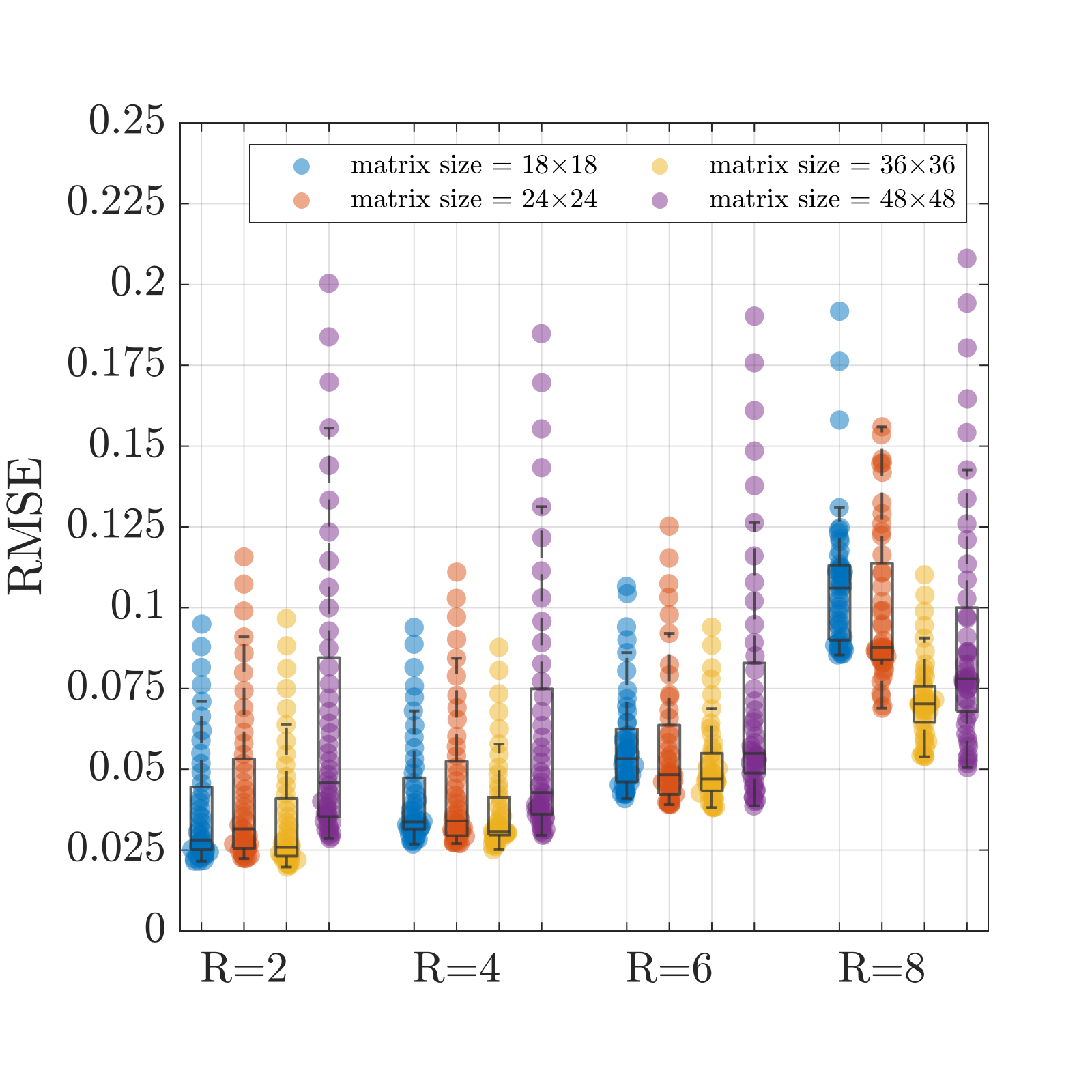


*Figure S2 - RMSE for different matrix sizes in the proposed method, at R=2, 4, 6 and 8. Each marker represents the RMSE from a single slice. Matrix sizes of 18x18 (blue), 24x24 (orange), 36x36 (yellow) and 48x48 (purple) were evaluated.*

Figure S3 shows, in the synthetic body dataset, the effect of different k-space matrix sizes across different acceleration factors in the proposed approach (TxLR). Interestingly, it appears that RMSE is minimized when the k-space matrix size is 36x36, across all acceleration factors. However, these differences are mostly small, and the changes in RMSE are largely driven by acceleration factor. The largest matrix size of 48x48 performs worst at all acceleration factors except R=8, where the smaller matrix sizes of 18x18 and 24x24 had the highest mean RMSE.


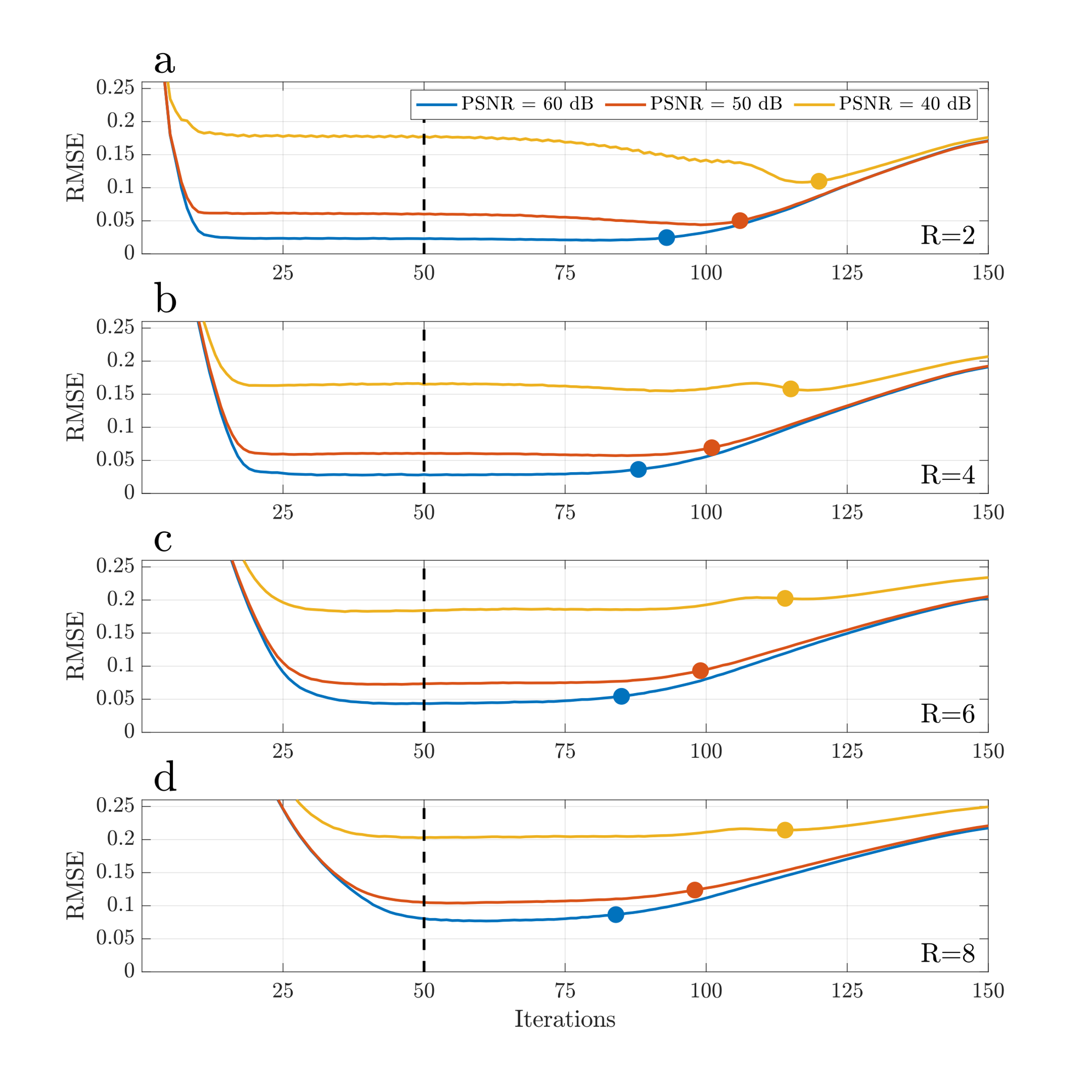


*Figure S3 - RMSE vs iteration count for the proposed method, evaluated at PSNRs of 40 (yellow), 50 (orange) and 60 dB (blue) and acceleration factors of (a) R=2, (b) R=4, (c) R=6, and (d) R=8. The RMSE at a fixed iteration count of 50 is denoted by the dashed black line, whereas the circles denote the number of iterations selected by the chi-square heuristic.*

An evaluation of the chi-square stopping heuristic is shown in Figure S4. The RMSE produced by a fixed iteration cutoff of 50 is compared to stopping iterations based on the chi-square heuristic, denoted by the circle markers. This was evaluated in the synthetic data on a central slice (z=24), at different PSNR levels (40 dB to 60 dB) and acceleration factors (R=2-8). We see that in these data, the choice to cut off iterations at 50 typically coincided with the beginning or middle of a plateau of the RMSE in all cases. In contrast, the chi-square heuristic typically selected the iteration parameters that corresponded the end of the plateau, in all cases, just before the RMSE begins to increase again as the reconstruction steps away from the local minimum. In either case, both fixed iterations and the chi-square heuristic were able to produce reconstructions very close to the local optimum, but the chi-square heuristic requires no *a priori* information (aside from receiver noise measurements) or guesswork.

## Justification for minimum measurement matrix for body relative transmit mapping

Using a single transverse slice from the middle of the synthetic body data set described in this work, the proton density was modulated by the transmit sensitives and Fourier transformed into k-space. Different measurement matrices around the centre of k-space were taken, including 10x10 and upto 48x48 in steps of 2. ESPIRiT(1) was used with varying kernel sizes of 3 to 10 to reconstruct transmit sensitivity maps. The RMS error in these reconstructed maps was compared to transmit sensitivity maps calculated from the full resolution data.

Figure S5 plots the RMS error for different kernel and matrix sizes, and figure S6 shows the error when using ESPIRiT to create a relative transmit sensitivity map.

*Figure S4: RMS error between ESPIRiT reconstructed transmit sensitivity maps and a reference created with fully sampled transmit sensitivity data*

**a**

**b**

*Figure S5: For an ESPIRiT reconstruction with a 24 x 24 matrix and 6 x 6 kernel, shown is a) the difference to the fully sampled reference, and b) the RMS error (of all 8 combined).*


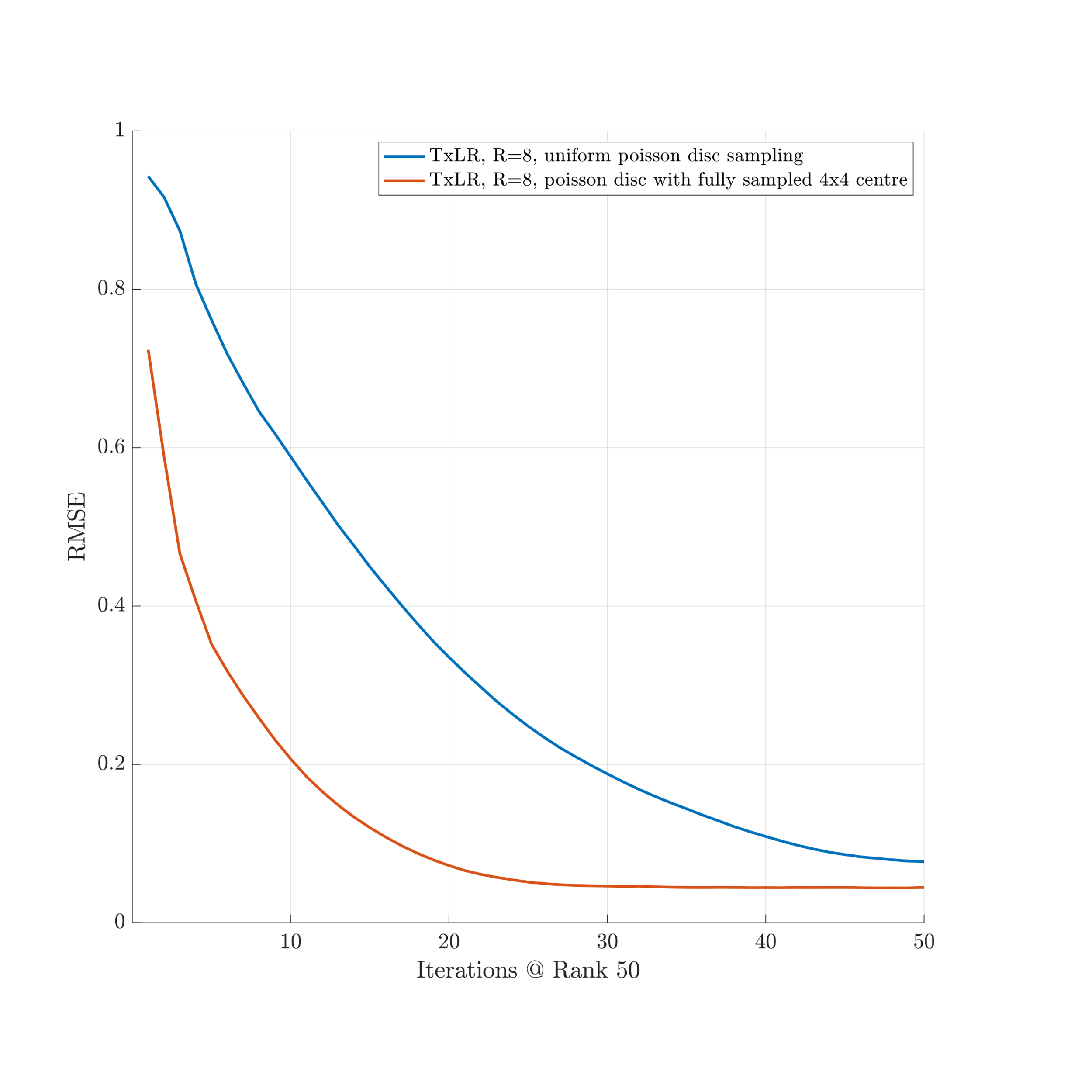


*Figure S6 - RMSE vs. iteration for uniform density sampling (blue), compared with a variable density sampling scheme that had uniform sampling in the periphery with a fully sampled 4x4 central k-space (orange). Both sampling schemes had an overall acceleration factor of R=8. The variable density scheme is shown to achieve lower minimum RMSE, and faster convergence.*

## Simulation of accelerated absolute B1+ mapping

Using a single transverse slice from the middle of the synthetic body data set described in this work, a pre-configuration pulse absolute B1+ mapping acquisition was simulated (2). Eight pairs of images were simulated, two for each transmit channel. The image pairs consisted of a small flip angle image ($PD(r)$), a complete T1 relaxation, followed by a pre-configuration pulse and image (${SS}_{pre}(r)$). The transmit voltage was set the same for all channels and ensured that the maximum pre-pulse flip angle was < 120°. These images were then Fourier transformed into k-space and a the central 24x24 was used to evaluate the reconstruction of B1+ maps for each channel. An acceleration factor of 8, with poison-disk under sampling and different under sampling masks for each of the 16 images. The images with the pre-configuration pulse were treated as additional transmit channels, making the transmit dimension 16.

The reconstructed k-spaces was Fourier transformed into images and combined in the receive dimension. Absolute maps were determined using the ratio of the two image :

| $\alpha(r)=acos(\frac{{SS}_{pre}(r)}{PD(r)})$ | (S1) |
| --- | --- |

The absolute B1+ maps were accurate where the pre-configuration pulse exceeded 40°, which is consistent with previous findings (2).

*Figure S7 - Eight times accelerated absolute B1+ mapping for an eight channel transmit system. The reconstructed absolute B1+ maps along with the error maps (difference to no acceleration) is shown.*
