# Supplementary material for: Accelerated calibrationless parallel transmit mapping using joint transmit and receive low-rank tensor completion
Source: Magn Reson Med. Author manuscript; Available in PMC 2021 Nov 1. (PMC7611890; doi:10.1002/mrm.28880)
Supplement: Appendix [file EMS136202-supplement-Appendix.pdf]

How to cite this article: Hess AT, Dragonu I, Chiew M. Accelerated calibrationless parallel transmit mapping using joint transmit and receive low-rank tensor completion. *Magn Reson Med*. 2021;00:1–14. https://doi.org/10.1002/mrm.28880

APPENDIX

We apply the ADMM to solve the non-convex optimization problem posed in Equation (6). Although convergence of the ADMM for non-convex problems is not guaranteed in general, many applications of the algorithm to non-convex problems have been demonstrated in the literature, with guarantees for certain classes of problems.[33] Empirically, we found the algorithm to work robustly when evaluated on the synthetic and real data investigated here.

We first re-cast Equation (6) as:

$$\min_{z} \frac{1}{2} \|Mz - \mathcal{D}\|_F^2 + \mathrm{I}_{r_1}\left(\mathcal{T}_{\mathcal{C}}\right) + \mathrm{I}_{r_2}\left(\mathcal{R}_{\mathcal{C}}\right)$$
$$\text{subject to: } \mathcal{T}_{\mathcal{C}} = U_1 T z$$
$$\text{subject to: } \mathcal{R}_{\mathcal{C}} = U_2 T z$$

where $\mathrm{I}_r(X)$ is the indicator function which evaluates to 0 if X has at most rank r, and ∞ otherwise. The augmented Lagrangian is formed as:

$$\mathcal{L}\left(z, \mathcal{T}_{\mathcal{C}}, \mathcal{R}_{\mathcal{C}}, y_1, y_2\right) = \frac{1}{2}\|Mz - \mathcal{D}\|_F^2 + \mathrm{I}_{r_1}\left(\mathcal{T}_{\mathcal{C}}\right) + \langle y_1, \mathcal{T}_{\mathcal{C}} - U_1 T z\rangle_F + \frac{\rho}{2}\|\mathcal{T}_{\mathcal{C}} - U_1 T z\|_F^2 + \mathrm{I}_{r_2}\left(\mathcal{R}_{\mathcal{C}}\right) + \langle y_2, \mathcal{R}_{\mathcal{C}} - U_2 T z\rangle_F + \frac{\rho}{2}\|\mathcal{R}_{\mathcal{C}} - U_2 T z\|_F^2$$

where $\langle , \rangle_F$ denotes the Frobenius inner product for matrices.

Using the scaled form of the ADMM (Equations (3.5)-(3.7) in Ref. 26), the first subproblems are solved by:

$$\mathcal{T}_{\mathcal{C}}^{n+1} = \operatorname*{argmin}_{\mathcal{T}_{\mathcal{C}}} \mathrm{I}_{r_1}\left(\mathcal{T}_{\mathcal{C}}\right) + \frac{\rho}{2}\left|\left|\mathcal{T}_{\mathcal{C}} - U_1 T z^{\boldsymbol{n}} - y_1^n\right|\right|_F^2$$

$$\mathcal{R}_{\mathcal{C}}^{n+1} = \operatorname*{argmin}_{\mathcal{R}_{\mathcal{C}}} \mathrm{I}_{r_2}\left(\mathcal{R}_{\mathcal{C}}\right) + \frac{\rho}{2}\left|\left|\mathcal{R}_{\mathcal{C}} - U_2 T z^{\boldsymbol{n}} - y_2^n\right|\right|_F^2$$

are solved by the singular value hard thresholding operations in lines (i) and (ii) of Equation (9).

Next, the z update in line (iii):

$$z^{n+1} = \operatorname*{argmin}_{z} \frac{1}{2}\|Mz - \mathcal{D}\|_F^2 + \frac{\rho}{2}\left\|\mathcal{T}_{\mathcal{C}}^{n+1} - U_1 T z + y_1^n\right\|_F^2 + \frac{\rho}{2}\left\|\mathcal{R}_{\mathcal{C}}^{n+1} - U_2 T z + y_2^n\right\|_F^2$$

can be rewritten as:

$$z^{n+1} = \operatorname*{argmin}_{z} \frac{1}{2}\left\|\begin{pmatrix} M \\ \rho U_1 T \\ \rho U_2 T \end{pmatrix} z - \begin{pmatrix} \mathcal{D} \\ \rho\left(\mathcal{T}_{\mathcal{C}}^{n+1} + y_1^n\right) \\ \rho\left(\mathcal{R}_{\mathcal{C}}^{n+1} + y_2^n\right) \end{pmatrix}\right\|_F^2$$

which is just a linear least squares problem, with a closed form solution which can be performed in a single step:

$$z^{n+1} = \left(M^H M + \rho T^H\left(U_1^H U_1 + U_2^H U_2\right) T\right)^{-1} \left(M^H \mathcal{D} + \rho T^H U_1^H\left(\mathcal{T}_{\mathcal{C}}^{n+1} + y_1^n\right) + \rho T^H U_2^H\left(\mathcal{R}_{\mathcal{C}}^{n+1} + y_2^n\right)\right)$$

The adjoint operators T^H, U_i^H are defined as follows: T^H, the adjoint block-Hankel operator, takes each column vector of the block-Hankel structured tensor, reshapes that into a 2D array, and places it back into its corresponding k-space location (eg, the dashed column vector in Figure 1B is transformed back to the dashed k-space 2D sub-array in Figure 1A), with overlapping values *summed* in place.[34] U_i^H, the adjoint unfolding operators are simply re-folding (reshaping and permuting) the matrix back into the original multi-dimensional array shape. The above closed form expression for z^{n+1} is significantly simplified by recognizing that $T^H U_1^H U_1 T$ and $T^H U_2^H U_2 T$ are diagonal operators and simply count the number of times any given k-space location is copied when constructing the block-Hankel structured tensor.

Finally, the dual variable updates for y_1, y_2 follow straightforwardly from their definition.

The ADMM algorithm implemented here also includes over-relaxation and variable penalty parameters.[26] Entries in red highlight the additional terms and steps beyond those shown in Equation (9). A complete description of the algorithm follows:

Initialize: $z^0 = 0$, $\mathbf{y}_{1,2}^0 = 0$, $\rho^0 = 10^{-6}$, $\tau = 1.1$, $\alpha = 1.5$

(i) $\mathcal{T}_{\mathcal{C}}^{n+1} = \Gamma_{r_1}\left(U_1 T z^n - y_1^n\right)$

(ii) $\mathcal{R}_{\mathcal{C}}^{n+1} = \Gamma_{r_2}\left(U_2 T z^n - y_2^n\right)$

(iii) $z^{n+1} = \operatorname*{argmin}_{z} \frac{1}{2}\|Mz - \mathcal{D}\|_F^2 +$

$$z^{n+1} = \operatorname*{argmin}_{z} \frac{1}{2}||Mz - \mathcal{D}||_F^2 + \frac{\rho^n}{2}\left|\left|\alpha \mathcal{T}_{\mathcal{C}}^{n+1} + (1-\alpha) U_1 T z^n - U_1 T z + y_1^n\right|\right|_F^2 + \frac{\rho^n}{2}\left|\left|\alpha \mathcal{R}_{\mathcal{C}}^{n+1} + (1-\alpha) U_2 T z^n - U_2 T z + y_2^n\right|\right|_F^2$$

(iv) $y_1^{n+1} = y_1^n + \alpha \mathcal{T}_{\mathcal{C}}^{n+1} + (1-\alpha) U_1 T z^n - U_1 T z^{n+1}$

(v) $y_2^{n+1} = y_2^n + \alpha \mathcal{R}_{\mathcal{C}}^{n+1} + (1-\alpha) U_2 T z^n - U_2 T z^{n+1}$

(vi)
$$\rho^{n+1} = \begin{cases} \tau \rho^n & \text{if} \left\|\begin{matrix} \mathcal{T}_{n+1}^{\mathcal{C}} - U_1 T z^{n+1} \\ \mathcal{R}_{n+1}^{\mathcal{C}} - U_2 T z^{n+1} \end{matrix}\right\|_F > 10\left\|\begin{matrix} \rho^n U_1 T\left(z^{n+1} - z^n\right) \\ \rho^n U_2 T\left(z^{n+1} - z^n\right) \end{matrix}\right\|_F \\ \rho^n/\tau & \text{if} \left\|\begin{matrix} \rho^n U_1 T\left(z^{n+1} - z^n\right) \\ \rho^n U_2 T\left(z^{n+1} - z^n\right) \end{matrix}\right\|_F > 10\left\|\begin{matrix} \mathcal{T}_{n+1}^{\mathcal{C}} - U_1 T z^{n+1} \\ \mathcal{R}_{n+1}^{\mathcal{C}} - U_2 T z^{n+1} \end{matrix}\right\|_F \\ \rho^n & \text{otherwise} \end{cases}$$
